# Patient-Perceived Overall Side Effect Bother at and After Cancer Treatment Discontinuation: An Analysis Using Commercial Cancer Trial Data

**DOI:** 10.1007/s43441-025-00906-8

**Published:** 2026-01-09

**Authors:** Jessica Roydhouse, Monique Breslin, Anne Zola, Ethan Basch, Melanie Calvert, David Cella, Mary Lou Smith, Gita Thanarajasingam, John Devin Peipert

**Affiliations:** 1 Menzies Institute for Medical Research, University of Tasmania, 15-17 Liverpool Street, Hobart, TAS 7000 Australia; 2 Kellogg School of Management, Northwestern University, Evanston, IL USA; 3 University of North Carolina Lineberger Comprehensive Cancer Center, Chapel Hill, NC USA; 4 Centre for Patient-Reported Outcomes Research, Birmingham Health Partners Centre for Regulatory Science and Innovation, National Institute for Health and Care Research (NIHR) Birmingham Biomedical Research Centre and NIHR Blood and Transplant Research Unit in Precision Cellular Therapeutics, University of Birmingham, Edgbaston, Birmingham, UK; 5 Feinberg School of Medicine, Northwestern University, Evanston, IL USA; 6 Research Advocacy Network, Plano, TX USA; 7 Division of Hematology, Mayo Clinic, Rochester, MN USA; 8 Centre for Patient-Reported Outcomes Research, Birmingham Health Partners Centre for Regulatory Science and Innovation, National Institute for Health and Care Research (NIHR) Birmingham Biomedical Research Centre, University of Birmingham, Edgbaston, Birmingham, UK

**Keywords:** Patient-reported outcome, Side effect, Adverse event, Tolerability, Clinical trial, Cancer

## Abstract

**Introduction:**

There is widespread interest among patients, clinicians, regulators and other constituents in post-treatment patient-reported cancer data. Side effect bother is a patient-reported outcome (PRO) that can capture an important aspect of tolerability. In this study, we examined side effect bother at cancer treatment discontinuation and post-discontinuation in commercial cancer trials. We sought to understand completion rates, the extent of bother and its association with other PROs.

**Materials and Methods:**

Data were evaluated from three trials in patients with solid tumours (renal cell carcinoma and breast cancer). Side effect bother was measured with the Functional Assessment of Chronic Illness Therapy (FACIT) GP5 item. Symptom items were drawn from FACIT and function items were drawn from the EQ-5D-3L. FACIT items, including the GP5, are on a 0–4 scale (higher = worse symptoms/bother), and were dichotomised as 0–1 (“low”) vs 2–4 (“moderate”). EQ-5D-3L items were characterised as no problems (1) and some problems (2–3). Descriptive and correlation analyses were conducted separately for each trial.

**Results:**

Among patients who received treatment, completion rates at discontinuation for most items were at least 70%, and 52% to 78% at follow up. More than 20% of patients had high side effect bother at and after discontinuation, and similar percentages were seen for symptom items and functioning problems. GP5 and most items were at least somewhat correlated (≥ 0.2 in nearly all evaluations).

**Discussion and Conclusions:**

Persistent side effect bother and symptomatic and functional detriments at and after discontinuation suggest capturing this information post-treatment can inform understanding of tolerability, particularly with improved PRO completion.

## Introduction

The acute effects of anti-cancer therapies such as chemotherapy are well-described in the literature [1], and treatment-related toxicity can persist well after treatment [2]. The side effects of newer therapies, such as targeted therapies or immunotherapies, can also seriously impact health-related quality of life [3]. Long-term follow-up of these therapies is also an important consideration when evaluating both adverse effects and health-related quality of life impacts [4]. For example, international regulatory guidance suggests a follow-up period of 15 years for chimeric antigen receptor T cell products (CAR-T) [5]. At the same time, there is a growing push towards the inclusion of patient-reported information about the impact of therapy in cancer trials, as evidenced by statements from regulatory bodies [6, 7]. Additionally, health-related quality of life plays a critical role in the decisions of health technology assessment bodies such as the National Institute for Health and Care Excellence (NICE) in the UK [8] and the Institute for Quality and Efficiency in Health Care (IQWiG) in Germany [9]. Payors value patient-reported outcome (PRO) data in oncology; in some cases, PROs have played an important role in decisions supporting products [10]. Payors also value post-progression data in oncology [10]. For example, a recent publication authored by individuals from IQWiG described post-progression data as “of paramount importance to patients and clinicians” [11]. Similarly, regulatory, academic, payor, and industry constituents at a 2020 US Food and Drug Administration (FDA) roundtable agreed that post-treatment PRO data in cancer clinical trials can provide important information about the persistence of symptomatic and functional impacts in patients treated for cancer [12].

Nonetheless, there are well-recognised challenges to the collection of post-treatment PRO data [12, 13]. Missing PRO data is a longstanding issue [14], and as pointed out in the literature, regulatory review [15, 16] has highlighted missing PRO data in regulatory submissions. Post-treatment PRO assessments were infrequent in a review of commercial clinical cancer trials, and when they occurred completion rates were on average 66% in either arm [17]. In contrast, median pre-treatment completion rates were over 90% and on-treatment completion rates close to 90% in commercial cancer clinical trials [18]. Similar findings of suboptimal post-discontinuation completion have been reported outside of the commercial clinical trial setting. Completion rates of 54–66% at treatment discontinuation were seen for the NRG’s GOG240 study [19], and response rates fell from 100% at randomization to 61% at 4-year follow-up and 46% at 7-year follow up in the PRO study component of SWOG S9133 [20].

Addressing and potentially improving the completion of post-treatment PRO data may be valuable [12, 13, 17]. Recommendations for post-treatment PRO data assessment include planning PRO assessments [12, 13, 17], using electronic PRO collection [13], and clearly defining specific PRO objectives [12, 13, 17]. Given concerns about long-term therapeutic impact, PRO objectives related to tolerability may be especially salient for post-treatment data collection. The low-grade but chronic adverse effect profiles of newer therapies [4] has highlighted a need for greater understanding of the longitudinal impact of treatment toxicity [21] and the inclusion of PROs [22] to aid in illuminating this. PROs related to tolerability include measures of symptomatic adverse events and overall side effect impact [6, 22]. In this study, we sought to characterise (1) the extent of overall side effect bother at the end of treatment and in post-treatment, (2) the association of overall side effect bother and symptom and function measures at the end of treatment and post-treatment, and (3) the completion rates for a single item for overall side effect bother at the end of treatment and post-treatment, using data from commercial cancer clinical trials.

## Methods

### Population and Data Sources

This study is part of a larger study evaluating the measurement properties of a single item for overall side effect bother, the GP5 item. GP5 is described in more detail below (see Measures). For this study, we accessed commercial clinical cancer trial data from the Vivli data sharing platform. All trials in our study had received HREC/IRB approval.

For this analysis, we worked with data from three solid tumour phase III (two renal cell, one breast) trials with an end of treatment and post-treatment follow up visit listed in the questionnaire file and where there was a variable indicating treatment discontinuation at a specific treatment cycle. The two renal cell trials evaluated identical agents, but one was a first-line trial and one was not. The breast cancer trial compared a combination of agents compared to one agent.

We used de-identified secondary data for this study and obtained confirmation that HREC/IRB approval was not required from our institutional committees/boards (Northwestern University and the University of Tasmania).

Within each trial, the study population was the safety population (i.e., patients who had received treatment) and who had discontinued for either disease progression or toxicity. We did not include patients who discontinued due to death as they could not be included in the survival follow-up, and we anticipated potentially high levels of missing data for the end of treatment visit. Patients who discontinued for other reasons, such as withdrawal of consent, would also be unlikely to have visits at the time points of interest, and were not included in our study population.

### Measures

We focused on five types of PRO items: (1) a side effect bother item (the primary PRO item of interest); (2) symptom items; (3) a bother item (not pertaining to side effect bother); (4) functional items; and (5) a global health item. Table 1 summarises the items and the data sources in which they are found for this analysis. Of note, the functional and global health items were only available for one data source (PALOMA) at the discontinuation visit only, and the bother item (not pertaining to side effect bother) was not available in two data sources (AXIS1, AXIS2). For the symptom items, two items (GP1, GP4) were available across all data sources and were the focus of the analysis for that broad PRO concept.

**Table 1:** Summary of PRO items and data source inclusion of PRO items

	Side effect bother	Symptom items	Bother items (other than side effect bother)	Functional items	Global health item
Measurement suite	Functional Assessment of Chronic Illness Therapy (FACIT)	FACIT	FACIT	EuroQol-5 Dimensions (EQ-5D) [30]	EuroQol-5 Dimensions (EQ-5D) [30]
PRO items in this concept	GP5: “I am bothered by the side effects of treatment”	GP1: “I have a lack of energy” GP4: “I have pain” HI7: “I feel fatigued” GP2: “I have nausea”	B5: “I am bothered by hair loss”	Mobility Self-care Usual Activities	Your health today
Recall period	7 days	7 days	7 days	Today	Today
Score range	0 (Not at all) to 4 (Very much)	0 (Not at all) to 4 (Very much)	0 (Not at all) to 4 (Very much)	0 (No problems) to 2 (Unable)	0 (worst)–100 (best)
Operationalisation in this study (Primary)	Low (0–1) vs Moderate-Severe (2–4) [31]	Low (0–1) vs Moderate-Severe (2–4)	Low (0–1) vs Moderate-Severe (2–4)	No Problems (0) vs Problems Present (1–2)	0–100
Operationalisation in this study (Sensitivity)	Low (0–2) vs Severe (3–4)	N/A	N/A	N/A	N/A
Data source inclusion of this concept					
*AXIS2*	GP5: FKSI-15 [32]	GP1, GP4, HI7: FKSI-15	Not included	EQ-5D-3L: Included at all time points	EQ-5D-3L: Included at all time points
*AXIS1*	GP5: FKSI-15 [32]	GP1, GP4, HI7: FKSI-15	Not included	EQ-5D-3L: Included at all time points	EQ-5D-3L: Included at all time points
*PALOMA*	GP5: FACT-G [33]	GP1, GP4, GP2: FACT-G	B5: FACT-B (Additional Concerns Scale)	EQ-5D-3L: Included at discontinuation visit only	EQ-5D-3L: Included at discontinuation visit only

### Analyses

Trials were described in terms of their clinical context (tumour type, agents being compared) and the scheduled end-of-treatment and first follow-up assessment as reported in their protocol. Population characteristics included age, baseline performance status (ECOG), and sex/gender (renal cell trials only, as the breast cancer trial was women-only).

For our analysis, we focused on three time points: (1) the end-of-treatment or discontinuation visit, at any point during the trial; (2) the discontinuation visit where it occurred earlier in the trial, defined here as within the first three cycles of treatment; and (3) the first follow-up visit after discontinuation. As there is relatively little known about the topic, we included an early discontinuation visit (as noted above, defined as within the first three cycles) to see if there were any differences in completion or severity.

To evaluate the extent of overall side effect impact at each time point, we used descriptive statistics (frequency and percentage [N, %]) to characterise GP5’s distribution, both overall and by reason for treatment discontinuation. Descriptive statistics (frequency and percentage) were used to characterise GP5 and all PRO items except the VAS, for which we used mean and standard deviation. The association between GP5 and the other measures described above was calculated in two ways. First, we evaluated the correlation at each time point using Spearman’s rho. Second, we evaluated dichotomised GP5 (less than moderate-severe: 0–1 vs moderate-severe: 2–4) against groups defined by symptom items and functional items through cross-tabulation. We focused on the items that were seen in all FACT measures (GP1, GP4); these were dichotomised as 0–1 (less than moderate-severe) vs 2–4 (moderate-severe). Where available, we also included the HI7 item (AXIS1, AXIS2 trials) and GP2 item (PALOMA trial). For the functional items in the EQ-5D, these were dichotomised as 1 (no problems) vs 2–3 (any problems). We expected that individuals with higher levels of symptoms, bother, or functional problems would be more likely to be in the moderate-severe (2–4) bother group. The descriptive and comparative analyses were repeated with GP5 dichotomised as 0–2 (less than severe) vs 3–4 (severe) for sensitivity analyses. Due to the exploratory nature of the study, available case analysis was used. We also examined within-person side effect bother between discontinuation and follow-up by estimating the number and percentage of people in each trial in change categories. This analysis was limited to people with complete responses for both the discontinuation and follow-up visits. We categorised within-person change as follows: (1) improved to less than moderate (i.e., went from 2–4 to < 2); (2) stayed less than moderate (i.e., did not increase from < 2); (3) stayed moderate (i.e., did not decrease from > 2); and (4) worsened to moderate (i.e., went from < 2 to ≥ 2).

Completion rates were calculated in several ways. First, we calculated the percentage of patients with a visit at the time points of interest. The denominator for this was the patients ‘expected’ to have the visit. For discontinuation visits, the denominator was patients who had received treatment (e.g., safety population) and had discontinued treatment for progression or toxicity. For the follow-up visit after treatment discontinuation, the denominator was restricted to patients who were listed as not having died per the survival file (e.g., would be eligible for a survival follow-up visit). Furthermore, for the two renal cell carcinoma trials, we were able to calculate the time between the survival/last contact date and discontinuation date and only considered patients with a time greater than or equal to the follow-up assessment period (e.g., patients for whom a follow-up visit could have been expected to have occur by the time of data cut). The numerator for all calculations was the occurrence of a discontinuation or follow-up visit, which was ascertained by whether the value for that visit appeared in the questionnaire dataset.

Second, we calculated the percentage of patients with a non-missing GP5 item at the time points of interest, with the ‘expected’ population as the denominator. Third, to facilitate comparison across items, we calculated the percentage of patients with non-missing GP5 and other items of interest at the time points of interest, with the number of patients with that visit as the denominator. We were interested in contextualising the GP5 item completion rate at these time points given earlier findings that GP5 completion at baseline may be lower than other items [23]. Finally, we also examined item completion by reasons for discontinuation, classified as progression/toxicity.

Each trial was analysed separately. All analyses were performed in R Studio.

## Results

### Included Trials and Study Population

The time frame for end of treatment assessments were not always specified; when specified, it was usually within one month of the last dose of treatment (Table 1). Follow-up assessments were highly variable, ranging from 28 days after the last dose to 6 months. When specified, windows were 7 days (Table 2). In the renal cell carcinoma studies, which recruited across both sexes, nearly all (> 70%) participants were male and had baseline ECOG scores of 0 or 1. In all trials, progression was the primary reason for discontinuation, and discontinuation due to toxicity/adverse events (AE) was infrequent (9–14% of patients across trials).

**Table 2:** Information about included trials and study population

	AXIS2	AXIS1	PALOMA
*Information from protocol*			
Cancer type	Metastatic renal cell carcinoma (2nd line)	Metastatic renal cell carcinoma (1st line)	ER+/HER2− advanced breast cancer
Treatments	Axitinib vs Sorafenib	Axitinib vs Sorafenib	Palbociclib + Letrozole vs Letrozole
End of treatment collection	At end of treatment or withdrawal	Unclear (assumed to be the same as AXIS1)	Within 4 weeks and 7 days of last dose and before new therapy
Follow-up collection	28 days after last dose	Unclear (assumed to be the same as AXIS1)	Every 6 months with a window of 7 days
*Information from clinicaltrials.gov*			
NCT number	NCT00678392	NCT00920816	NCT01740427
Trial phase	Phase 3	Phase 3	Phase 3
ITT population	723	288 (first-line participants only)	666
Safety population (from clinicaltrials.gov)	714	261 (first-line participants only, and no participants from China)	666
Duration of treatment phase	Up to 3 years from treatment start to follow-up	Up to Week 107 (for first-line participants)	Up to 2.5 years (end of treatment)
PRO measures/endpoints used (from clinicaltrials.gov)	FKSI-15, EQ-5D-3L	FKSI-15, EQ-5D-3L	FACT-B, EQ-5D-3L
*Information from study analyses*			
Age: Mean (standard deviation)*	59.56 (10.46)	57.25 (10.71)	59.87 (11.26)
Sex*			
Male	265 (70.1)	80 (74.1)	321 (100.0)^
Female	113 (29.9)	28 (25.9)	
Baseline ECOG score*^#^			
ECOG = 0	204 (54.0)	68 (63.0)	159 (49.5)
ECOG = 1	174 (46.0)	40 (37.0)	155 (48.3)
ECOG = 2	0 (0.0)	0 (0.0)	7 (2.2)
Discontinuation Reason*			
Progression	324 (85.7)	98 (90.7)	288 (89.7)
Toxicity	54 (14.3)	10 (9.3)	33 (10.3)
Health Status (EQ-5D VAS^+^): Mean (standard deviation)*	61.61 (20.75)	65.67 (20.76)	69.78 (20.43)

*Summary is from discontinuation visit due to larger sample size; ^Analyses or eligibility criteria restricted to women only; ^#^ECOG: Eastern Cooperative Oncology Group; ^+^VAS: Visual Analogue Scale

### GP5 and Other PROs at End of Treatment and Follow-Up

At the treatment discontinuation visit, the percentage of patients with GP5 scores of at least 2 (“moderate to severe bother”) ranged from 30 to 57% (Table 3). At discontinuation, at least 20% of patients reported problems of some kind as reported on EQ-5D-3L; lack of energy was reported by at least 50% of patients in all trials, and pain by at least 40% (Table 3). However, the average VAS score was at least 60/100 (Table 3). When early discontinuation was considered, the percentage of patients with GP5 scores of at least 2 was at least 50%; at least 50% reported scores of at least 2 for lack of energy and at least 50% reported had scores of 2 or higher for pain (Table 3). In the breast cancer trial, < 30% of patients had scores of at least 2 on hair loss bother at all time points, and < 20% had scores of at least 2 on nausea at all time points (Table 3). At follow-up, at least 20% of patients in both AXIS trials had scores of 3 or higher for GP1 (Lack of Energy), compared to 13% in the PALOMA trial. However, at least 30% in each trial had scores of 3 or higher for GP4 (Pain). At least 10% in each trial had scores of 3 or higher for GP5, though numbers were very small in the AXIS1 trial (n = 4) (Table 3).

**Table 3:** PRO scores after treatment discontinuation: presence of problems/moderate symptoms or bother^#^

Trial	AXIS2	AXIS1	PALOMA
*Scores from discontinuation visit (any time after treatment)*			
Usual activities: any problems*	187 (64.9)	55 (64.7)	141 (50.9)
Self-care: any problems*	86 (29.9)	29 (34.5)	58 (21.2)
Mobility: any problems*	146 (50.5)	52 (61.9)	125 (45.5)
VAS: mean (SD)	61.61 (20.75)	65.67 (20.76)	69.78 (20.43)
GP1 Lack energy: moderate-severe (2–4)	174 (60.6)	53 (61.6)	139 (50.5)
GP1 Lack energy: severe (3–4)	69 (24.0)	22 (25.6)	59 (21.5)
GP4 Pain: moderate-severe (2–4)	146 (51.0)	45 (52.3)	119 (43.8)
GP4 Pain: severe (3–4)	67 (23.4)	17 (19.8)	55 (20.2)
GP5 Side effect bother: moderate-severe (2–4)	160 (56.7)	40 (47.1)	81 (29.7)
GP5 Side effect bother: severe (3–4)	67 (23.8)	13 (15.3)	32 (11.7)
HI7 Fatigue: moderate-severe (2–4)	160 (55.9)	50 (58.1)	NA
HI7 Fatigue: severe (3–4)	73 (25.5)	18 (20.9)	NA
GP2 Nausea: moderate-severe (2–4)	NA	NA	42 (15.4)
GP2 Nausea: severe (3–4)	NA	NA	12 (4.4)
B5 Hair loss bother: moderate-severe (2–4)	NA	NA	74 (27.4)
B5 Hair loss bother: severe (3–4)	NA	NA	43 (15.9)
*Scores from early discontinuation visit (within first three cycles)*			
Usual activities: any problems*	71 (65.7)	4 (44.4)	40 (67.8)
Self-care: any problems*	37 (34.3)	1 (12.5)	19 (33.3)
Mobility: any problems*	61 (56.0)	4 (50.0)	33 (56.9)
VAS: mean (SD)	58.85 (22.45)	73.00 (13.37)	59.59 (21.89)
GP1 Lack energy: moderate-severe (2–4)	63 (58.3)	5 (55.6)	41 (70.7)
GP1 Lack energy: severe (3–4)	30 (27.8)	3 (33.3)	20 (34.5)
GP4 Pain: moderate-severe (2–4)	60 (56.1)	7 (77.8)	32 (55.2)
GP4 Pain: severe (3–4)	34 (31.8)	3 (33.3)	16 (27.6)
GP5 Side effect bother: moderate-severe (2–4)	69 (65.7)	4 (44.4)	21 (36.2)
GP5 Side effect bother: severe (3–4)	36 (34.3)	0 (0.0)	10 (17.2)
HI7 Fatigue: moderate-severe (2–4)	59 (55.1)	6 (66.7)	NA
HI7 Fatigue: severe (3–4)	32 (29.9)	2 (22.2)	NA
GP2 Nausea: moderate-severe (2–4)	NA	NA	11 (19.0)
GP2 Nausea: severe (3–4)	NA	NA	2 (3.4)
B5 Hair loss bother: moderate-severe (2–4)	NA	NA	16 (28.6)
B5 Hair loss bother: severe (3–4)	NA	NA	12 (21.4)
*Scores from first follow-up visit after treatment discontinuation*			
Usual activities: any problems*	44 (46.8)	17 (54.8)	NA
Self-care: any problems*	12 (12.9)	10 (32.3)	NA
Mobility: any problems*	33 (35.1)	16 (51.6)	NA
VAS: mean (SD)	69.58 (19.40)	66.39 (21.09)	NA
GP1 Lack energy: moderate-severe (2–4)	42 (43.3)	16 (51.6)	17 (37.0)
GP1 Lack energy: severe (3–4)	21 (21.6)	7 (22.6)	6 (13.0)
GP4 Pain: moderate-severe (2–4)	29 (30.2)	11 (35.5)	14 (30.4)
GP4 Pain: severe (3–4)	10 (10.4)	5 (16.1)	4 (8.7)
GP5 Side effect bother: moderate-severe (2–4)	32 (34.4)	12 (38.7)	11 (24.4)
GP5 Side effect bother: severe (3–4)	14 (15.1)	4 (12.9)	6 (13.3)
HI7 Fatigue: moderate-severe (2–4)	42 (43.3)	16 (51.6)	NA
HI7 Fatigue: severe (3–4)	21 (21.6)	7 (22.6)	NA
GP2 Nausea: moderate-severe (2–4)	NA	NA	5 (10.9)
GP2 Nausea: severe (3–4)	NA	NA	3 (6.5)
B5 Hair loss bother: moderate-severe (2–4)	NA	NA	8 (17.4)
B5 Hair loss bother: severe (3–4)	NA	NA	6 (13.0)

*Scores of 2–3 (range is 1–3); ^#^N(%) unless otherwise indicated

Analyses considering scores of at least 3 on all FACIT/FACT items, including the GP5, showed that a much smaller proportion of patients had scores of 3 or 4 on these items. For example, at the discontinuation visit, 47.1% patients had scores of 2–4 on the GP5 item, but 15.3% had scores of 3–4 on the item (Table 3). This pattern was seen at other time points (Table 3). A similar pattern was seen for the other FACIT/FACT items (Table 3).

**Fig. 1:**
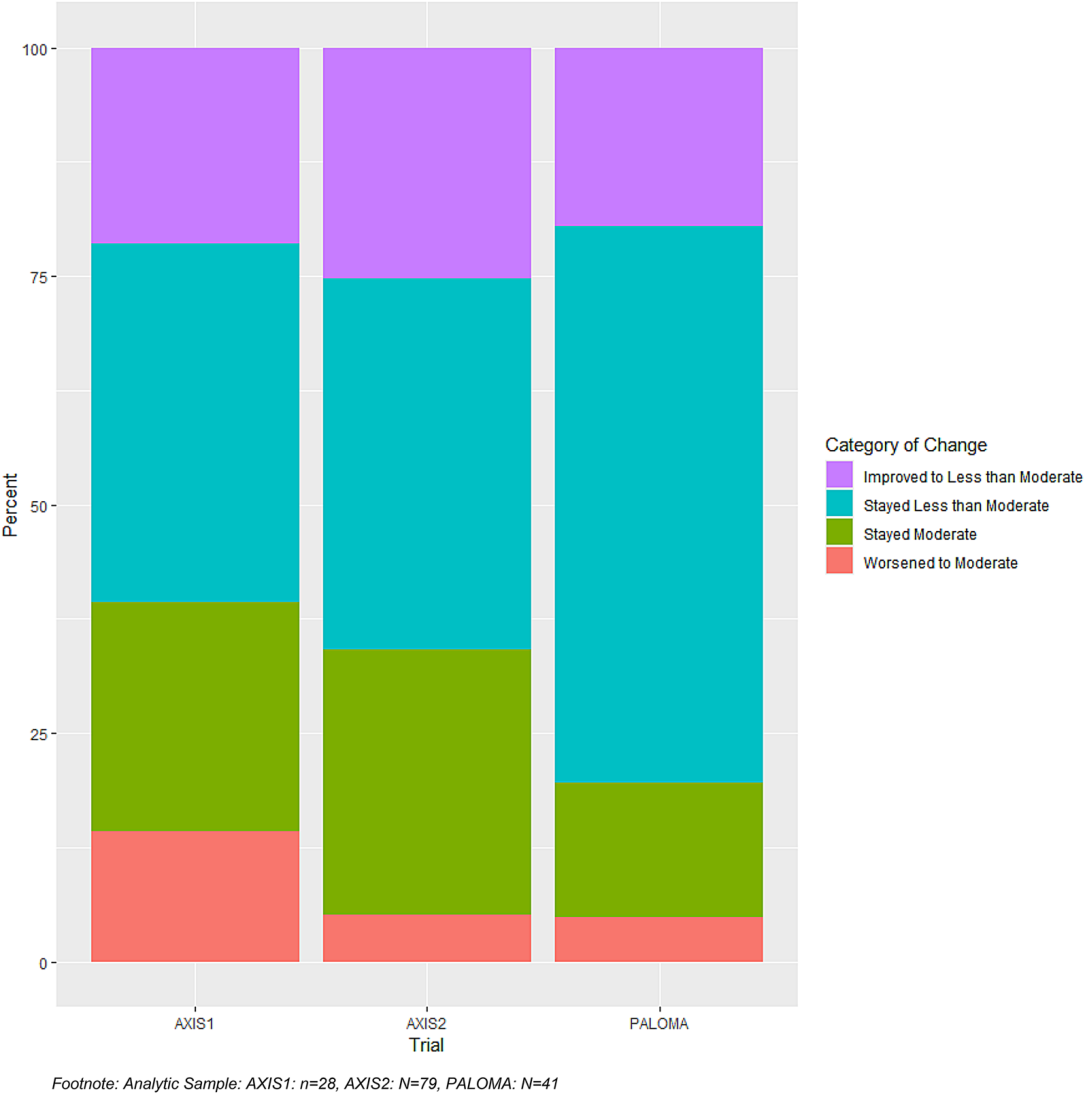
Within-person GP5 score change: discontinuation visit to follow-up

When within-person score change was examined, at least 20% patients in each trial improved to less than moderate (scores < 2) from scores of moderate-severe (scores 2–4). In all three trials, the category with the largest percentage of patients was ‘Stayed Less than Moderate.’ Relatively few patients worsened between discontinuation and follow-up, and > 15% in each trial had persistent moderate-severe bother (“stayed moderate”) (Fig. 1).

### GP5: Correlation with Other Items

Correlations of at least 0.3 were seen between GP5 and most items at the discontinuation visit (Table 4). The exceptions were self-care (0.14 and 0.19 in two trials), pain (0.29 in one trial), usual activities (0.29 in one trial) and hair loss bother (0.29 in one trial). At the early discontinuation and follow-up visits, correlations were generally 0.2 or higher for most items (Table 4).

**Table 4:** Correlation*: GP5 and Other Items

Trial	AXIS2 GP5 N = 282	AXIS1 GP5 N = 85	PALOMA GP5 N = 273
*Discontinuation visit*			
Usual activities	0.30	0.29	0.35
Self-care	0.19	0.14	0.35
Mobility	0.30	0.30	0.34
VAS	− 0.32	− 0.44	− 0.37
GP1 Lack energy	0.37	0.36	0.48
GP4 Pain	0.42	0.29	0.41
HI7 Fatigue	0.36	0.39	NA
GP2 Nausea	NA	NA	0.37
B5 Hair loss bother	NA	NA	0.29

Trial	AXIS2 GP5 N = 155	AXIS1 GP5 N = 14	PALOMA GP5 N = 71
*Discontinuation visit (early)*			
Usual activities	0.25	0.59	0.22
Self-care	0.18	0.36	0.43
Mobility	0.33	0.35	0.30
VAS	− 0.32	− 0.20	− 0.37
GP1 Lack energy	0.23	0.69	0.45
GP4 Pain	0.40	− 0.48	0.26
HI7 Fatigue	0.23	0.27	NA
GP2 Nausea	NA	NA	0.45
B5 Hair loss bother	NA	NA	0.23

Trial	AXIS2 GP5 N = 171	AXIS1 GP5 N = 40	PALOMA GP5 N = 86
*Follow up*			
Usual activities	0.35	0.56	NA
Self-care	0.21	0.60	NA
Mobility	0.42	0.60	NA
VAS	− 0.33	− 0.48	NA
GP1 Lack energy	0.38	0.49	0.55
GP4 pain	0.37	0.59	0.62
HI7 Fatigue	0.42	0.60	NA
GP2 Nausea	NA	NA	0.52
B5 Hair loss bother	NA	NA	0.38

*Spearman’s rho

### Presence of Problems, Global Health Scores and Symptom Severity Across GP5 Categories

**Fig. 2:**
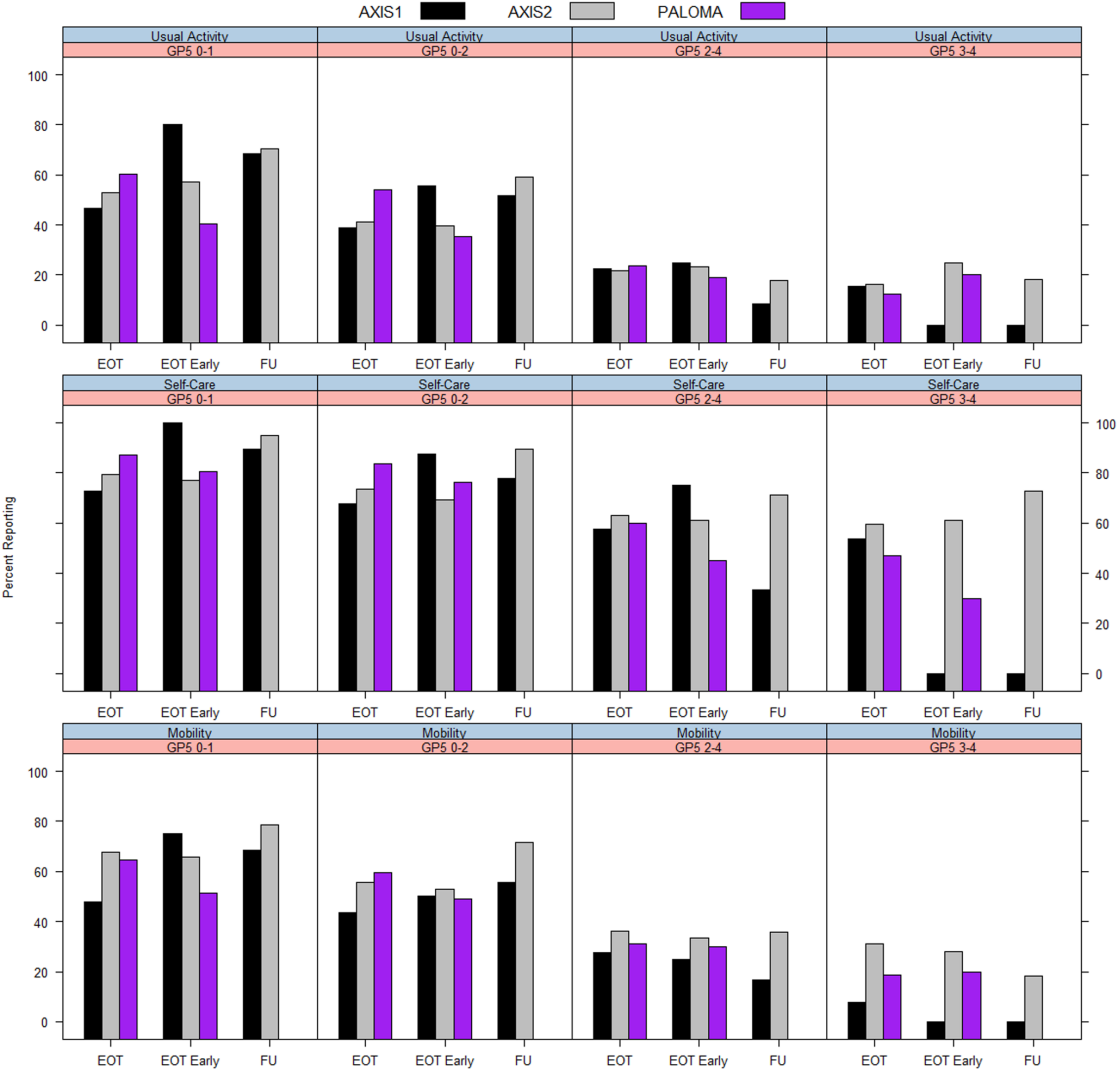
Percentage of patients with ‘no problems’ on functioning items across GP5 categories

Regardless of the time point, there were higher percentages of patients reporting ‘No Problems’ for usual activities or mobility when GP5 was categorised as 0–1 (less than moderate-severe) or 0–2 (less than severe). The results were similar but less pronounced for self-care, where there were still high percentages of patients with ‘No Problems’ for this item yet moderate-severe (2–4) or severe (3–4) bother on GP5 (Fig. 2; Table 5). The mean VAS score decreased steadily with each GP5 category. For example, for AXIS2 the mean VAS score at discontinuation was 68.50 (SD 18.45) for the GP5 category of 0–1, compared to 64.63 (SD = 19.32) for 0–2, 56.31 (SD = 20.56) for 2–4 and 51.88 (SD = 21.45) for 3–4 (Table 5). Similar results were seen for other time points and in the other trials.

**Table 5:** Functioning and global health items within strata of GP5

		AXIS2				AXIS1				PALOMA			
GP5 group		0–1	0–2	2–4	3–4	0–1	0–2	2–4	3–4	0–1	0–2	2–4	3–4
EOT	PRO score	*N* = *122*	*N* = *215*	*N* = *160*	*N* = *67*	*N* = *45*	*N* = *72*	*N* = *40*	*N* = *13*	*N* = *192*	*N* = *241*	*N* = *81*	*N* = *32*
*Usual activities*	1	64 (52.9)	88 (41.1)	35 (21.9)	11 (16.4)	21 (46.7)	28 (38.9)	9 (22.5)	2 (15.4)	115 (60.2)	130 (54.2)	19 (23.5)	4 (12.5)
*Self-care*	1	96 (79.3)	157 (73.4)	101 (63.1)	40 (59.7)	32 (72.7)	48 (67.6)	23 (57.5)	7 (53.8)	165 (87.3)	198 (83.5)	48 (60.0)	15 (46.9)
*Mobility*	1	82 (67.8)	119 (55.6)	58 (36.2)	21 (31.3)	21 (47.7)	31 (43.7)	11 (27.5)	1 (7.7)	123 (64.7)	142 (59.7)	25 (31.2)	6 (18.8)
*VAS*	Mean (SD)	68.50 (18.45)	64.63 (19.32)	56.31 (20.56)	51.88 (21.45)	72.53 (17.83)	69.82 (17.24)	57.95 (21.31)	42.69 (24.17)	73.64 (19.55)	71.84 (19.74)	60.77 (19.89)	54.68 (19.95)
EOT early	PRO score	*N* = *36*	*N* = *69*	*N* = *69*	*N* = *36*	*N* = *5*	*N* = *9*	*N* = *4*	*N* = *0*	*N* = *37*	*N* = *48*	*N* = *21*	*N* = *10*
*Usual activities*	1	20 (57.1)	27 (39.7)	16 (23.2)	9 (25.0)	4 (80.0)	5 (55.6)	1 (25.0)	0	15 (40.5)	17 (35.4)	4 (19.0)	2 (20.0)
*Self-care*	1	27 (77.1)	47 (69.1)	42 (60.9)	22 (61.1)	4 (100.0)	7 (87.5)	3 (75.0)	0	29 (80.6)	35 (76.1)	9 (45.0)	3 (30.0)
*Mobility*	1	23 (65.7)	36 (52.9)	23 (33.3)	10 (27.8)	3 (75.0)	4 (50.0)	1 (25.0)	0	19 (51.4)	23 (48.9)	6 (30.0)	2 (20.0)
*VAS*	Mean (SD)	68.33 (21.60)	61.58 (22.55)	53.42 (21.12)	52.69 (21.03)	72.60 (14.93)	73.00 (13.37)	73.50 (13.38)	0	64.42 (21.30)	61.89 (22.58)	51.76 (21.46)	49.70 (16.79)
FU	PRO score	*N* = *61*	*N* = *79*	*N* = *32*	*N* = *14*	*N* = *19*	*N* = *27*	*N* = *12*	*N* = *4*	*NA*	*NA*	*NA*	*NA*
*Usual activities*	1	43 (70.5)	46 (59.0)	5 (17.9)	2 (18.2)	13 (68.4)	14 (51.9)	1 (8.3)	0	NA	NA	NA	NA
*Self-care*	1	57 (95.0)	69 (89.6)	20 (71.4)	8 (72.7)	17 (89.5)	21 (77.8)	4 (33.3)	0				
*Mobility*	1	48 (78.7)	56 (71.8)	10 (35.7)	2 (18.2)	13 (68.4)	15 (55.6)	2 (16.7)	0	NA	NA	NA	NA
*VAS*	Mean (SD)	73.46 (20.59)	71.76 (19.17)	62.21 (15.02)	56.55 (18.13)	75.37 (17.33)	68.74 (19.74)	52.17 (19.03)	50.50 (26.20)	NA	NA	NA	NA

**Fig. 3:**
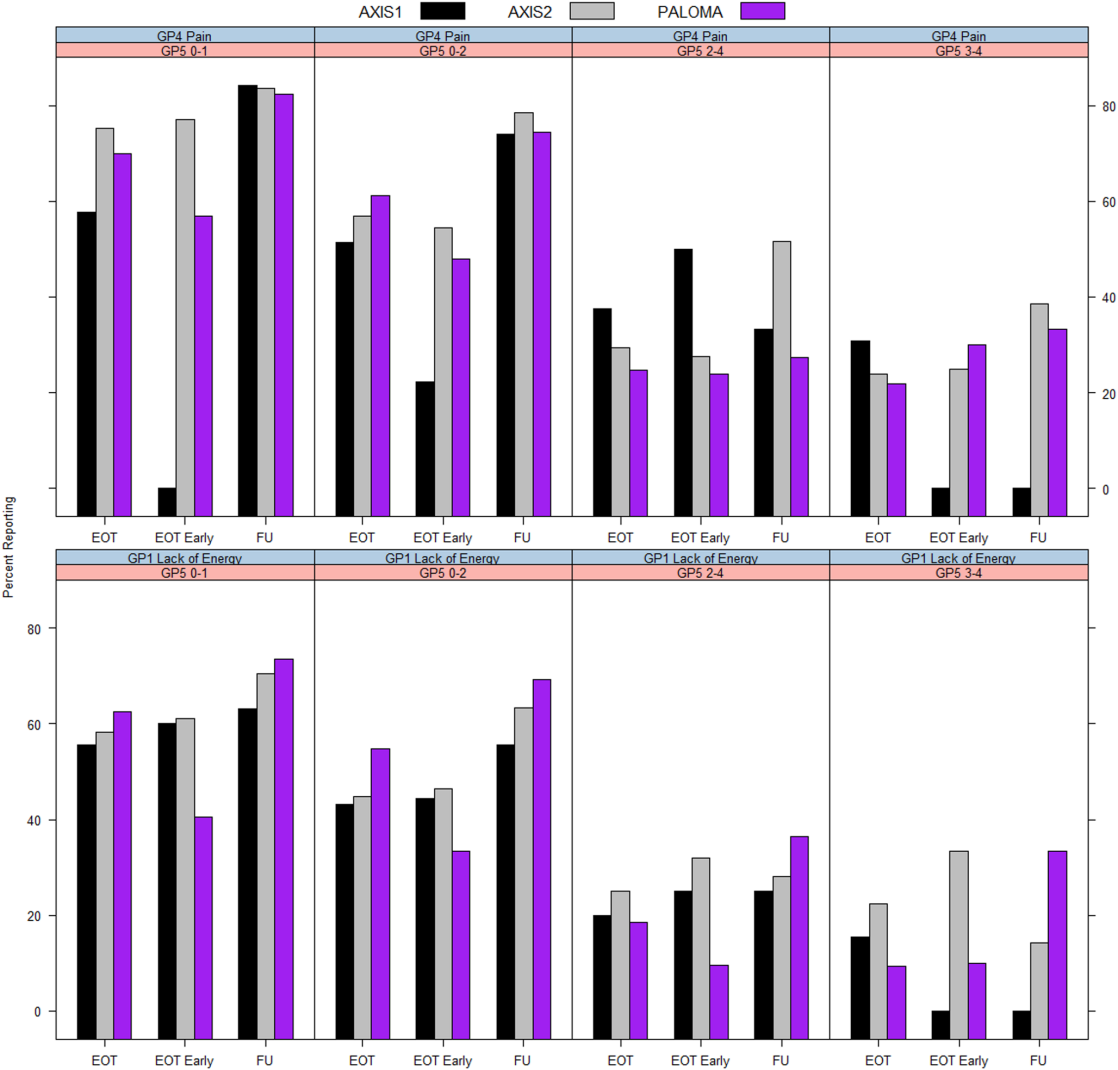
Percentage of patients with ‘less than moderate-severe symptoms’ across GP5 categories

A similar pattern was seen for less than moderate-severe (0–1) symptoms (GP1 Lack of Energy, GP4 Pain) (Fig. 3; Table 6). For example, the percentage of patients who had scores of 0–1 (less than moderate-severe) on GP1 Lack of Energy in PALOMA at discontinuation was 62.5% for GP5 0–1, compared to 54.8% for GP5 0–2, 18.5% for GP5 2–4 and 9.4% for GP5 3–4 (Table 6). Similar findings were seen for other symptom items such as HI7 Fatigue (Appendix Table 8), GP2 Nausea (Appendix Table 9), and the bother item B5 Hair Loss Bother (Appendix Table 9).

**Table 6:** Common symptom items within strata of GP5

		AXIS2				AXIS1				PALOMA			
GP5 group		GP5 0–1	GP5 0–2	GP5 2–4	GP5 3–4	GP5 0–1	GP5 0–2	GP5 2–4	GP5 3–4	GP5 0–1	GP5 0–2	GP5 2–4	GP5 3–4
Discontinuation (any)	PRO score	*N* = *122*	*N* = *215*	*N* = *160*	*N* = *67*	*N* = *45*	*N* = *72*	*N* = *40*	*N* = *13*	*N* = *192*	*N* = *241*	*N* = *81*	*N* = *32*
*GP1 Lack energy*	0–1	71 (58.2)	96 (44.7)	40 (25.0)	15 (22.4)	25 (55.6)	31 (43.1)	8 (20.0)	2 (15.4)	120 (62.5)	132 (54.8)	15 (18.5)	3 (9.4)
*GP4 Pain*	0–1	91 (75.2)	122 (57.0)	47 (29.4)	16 (23.9)	26 (57.8)	37 (51.4)	15 (37.5)	4 (30.8)	133 (70.0)	146 (61.1)	20 (24.7)	7 (21.9)
Discontinuation (early)	PRO score	*N* = *36*	*N* = *69*	*N* = *69*	*N* = *36*	*N* = *5*	*N* = *9*	*N* = *4*	*N* = *0*	*N* = *37*	*N* = *48*	*N* = *21*	*N* = *10*
*GP1 Lack energy*	0–1	22 (61.1)	32 (46.4)	22 (31.9)	12 (33.3)	3 (60.0)	4 (44.4)	1 (25.0)	0	15 (40.5)	16 (33.3)	2 (9.5)	1 (10.0)
*GP4 Pain*	0–1	27 (77.1)	37 (54.4)	19 (27.5)	9 (25.0)	0 (0.0)	2 (22.2)	2 (50.0)	0	21 (56.8)	23 (47.9)	5 (23.8)	3 (30.0)
Follow-up	PRO score	*N* = *61*	*N* = *79*	*N* = *32*	*N* = *14*	*N* = *19*	*N* = *27*	*N* = *12*	*N* = *4*	*N* = *34*	*N* = *39*	*N* = *11*	*N* = *6*
*GP1 Lack energy*	0–1	43 (70.5)	50 (63.3)	9 (28.1)	2 (14.3)	12 (63.2)	15 (55.6)	3 (25.0)	0	25 (73.5)	27 (69.2)	4 (36.4)	2 (33.3)
*GP4 Pain*	0–1	51 (83.6)	62 (78.5)	16 (51.6)	5 (38.5)	16 (84.2)	20 (74.1)	4 (33.3)	0	28 (82.4)	29 (74.4)	3 (27.3)	2 (33.3)

### Visit Occurrence and GP5 Completion as a Percentage of ‘Expected’

In the three included trials, nearly all (> 80%) patients had a discontinuation visit when expected (Appendix Table 10). GP5 completion, when considering the ‘expected’ patients as the denominator, ranged from 70 to 80% for discontinuation visits at any time, but 56–80% for early discontinuation visits. Follow-up visit occurrence ranged from 42 to 93% of expected and GP5 completion, when considering the ‘expected’ patients as the denominator, was 22–69%.

### GP5 and PRO Completion Rates

Completion rates were higher at discontinuation than follow-up for all studies (Table 7). For discontinuation visits at any time, completion rates for most items were at least 75% and 76–85% for GP5 (Table 7). Completion rates at early discontinuation visits ranged from at least 57% to 83% for most items, and 64–82% for GP5 (Table 7). At follow-up, completion rates for most items were 54–78%, and 52–78% for GP5 (Table 7).

**Table 7:** Completion rates at different visits*

	AXIS2	AXIS1	PALOMA
*Discontinuation (any)*	*N* = *378*	*N* = *108*	*N* = *321*
Usual activities	288 (76.2)	85 (78.7)	277 (86.3)
Self-care	288 (76.2)	84 (77.8)	274 (85.4)
Mobility	289 (76.5)	84 (77.8)	275 (85.7)
VAS	286 (75.7)	85 (78.7)	273 (85.0)
GP1 Lack energy	287 (75.9)	86 (79.6)	275 (85.7)
GP4 Pain	286 (75.7)	86 (79.6)	272 (84.7)
GP5 Side effect bother	282 (74.6)	85 (78.7)	273 (85.0)
HI7 Fatigue	286 (75.7)	86 (79.6)	NA
GP2 Nausea	NA	NA	273 (85.0)
B5 Hair loss bother	NA	NA	270 (84.1)
*Discontinuation (early)*	*N* = *155*	*N* = *14*	*N* = *71*
Usual activities	108 (69.7)	9 (64.3)	59 (83.1)
Self-care	108 (69.7)	8 (57.1)	57 (80.3)
Mobility	109 (70.3)	8 (57.1)	58 (81.7)
VAS	109 (70.3)	9 (64.3)	58 (81.7)
GP1 Lack energy	108 (69.7)	9 (64.3)	58 (81.7)
GP4 Pain	107 (69.0)	9 (64.3)	58 (81.7)
GP5 Side effect bother	105 (67.7)	9 (64.3)	58 (81.7)
HI7 Fatigue	107 (69.0)	9 (64.3)	NA
GP2 Nausea	NA	NA	58 (81.7)
B5 Hair loss bother	NA	NA	56 (78.9)
*Follow-up*	*N* = *171*	*N* = *40*	*N* = *86*
Usual activities	94 (55.0)	31 (77.5)	NA
Self-care	93 (54.4)	31 (77.5)	NA
Mobility	94 (55.0)	31 (77.5)	NA
VAS	92 (53.8)	31 (77.5)	NA
GP1 Lack energy	97 (56.7)	31 (77.5)	46 (53.5)
GP4 Pain	96 (56.1)	31 (77.5)	46 (53.5)
GP5 Side effect bother	93 (54.4)	31 (77.5)	45 (52.3)
HI7 Fatigue	97 (56.7)	31 (77.5)	NA
GP2 Nausea	NA	NA	46 (53.5)
B5 Hair loss bother	NA	NA	46 (53.5)

*Number with visit as denominator

At nearly all time points and for all items, completion rates were higher when patients discontinued for progression as opposed to toxicity (Appendix Table 11). The exception was the AXIS1 trial, however at most 10 patients discontinued for toxicity at these time points. Within reasons for discontinuation, completion rates were similar between items at all time points, with no evidence of lower GP5 completion (Appendix Table 11). The exception was AXIS2 at progression, where, among patients discontinuing for toxicity (n = 25), completion for GP5 was 44% (n = 11), compared to 52% (n = 13) (Appendix Table 11).

## Discussion

Moderate-severe levels of side effect bother and symptoms (scores of 2 or higher out of 4) were seen by at least 20% of patients in follow-up, underscoring the potential usefulness of post-treatment data collection. Similar results were seen at the discontinuation visit, whether it was examined at any point in the trial or when discontinuation visits only in the first three months were considered. Although fewer patients had severe bother or symptoms (scores of 3 or higher out of 4), patients with moderate side effect bother may carry residual symptoms long after treatment discontinuation. Our study also demonstrated associations between the GP5 item and functioning, symptom, and global health PROs, providing further evidence in support of the item’s psychometric validity. PRO completion, including completion of the GP5 item, was variable across studies and lower at follow-up than end-of-treatment. There was no evidence of lower completion of the GP5 item, compared to other PRO items, at any of these time points.

Like previous studies, we found low rates of post-treatment patient-reported outcome measure (PROM) completion, particularly at follow-up visits [17, 20]. To the best of our knowledge, there are no standard completion rate calculation metrics for these visits. Not surprisingly, we found lower completion if we considered the denominator to be patients for whom the visit was ‘expected,’ rather than those who had the visit. There may be different reasons why patients do not attend discontinuation or follow-up visits, and it is not clear if the number of ‘expected’ is a reasonable denominator for calculating completion rates at these visits. Development of guidelines and standards would be beneficial and aid comparison across trials. In any case, our findings of comparatively lower completion at follow-up compared to discontinuation were robust regardless of the denominator used.

It is somewhat concerning, though perhaps not surprising, that completion rates were comparatively low for discontinuation due to toxicity compared to discontinuation due to progression. If patients who discontinued due to toxicity had higher levels of side effect bother, our findings may be underestimating the level of side effect impact at discontinuation and post-treatment. However, discontinuation due to toxicity does not necessarily mean that symptomatic adverse events were a key factor; for example, trial protocols may indicate discontinuation due to lab-based toxicity. The number of patients discontinuing due to toxicity was generally small across the trials, posing challenges for definitive conclusions.

As there is interest in capturing patient perspectives at the end of treatment, for example through exit interviews [24, 25] or evaluation of how valuable patients perceived treatment to be [26], working to improve PROM completion at discontinuation is worthwhile. Additionally, collection of the reasons for missing PRO data is particularly important after treatment [12]. Earlier work has identified that GP5 completion at baseline may be lower than that for other PROs [23]; however, we found no evidence of this at either the discontinuation or post-discontinuation visits.

Another consideration for discontinuation and post-discontinuation PROM collection is the timing of follow-up PRO assessments, as schedules may affect outcomes [27–29]. Consistent with the literature [13, 17], we found PRO assessment timing at and after discontinuation to be heterogeneous. Focusing on assessment at specific post-discontinuation time points [12] or following the pre-discontinuation assessment schedule until a new treatment is taken up [13] have been suggested. In any case, if PRO collection will be undertaken post-discontinuation, working with trialists to optimise completion can help in minimising missing data and maximising data value and interpretability. Furthermore, well-designed objectives can help determine the suitability of post-discontinuation completion as well as the appropriate assessment schedule.

In our study, we found persistent symptoms and side effect bother after treatment discontinuation. Attributing PROs to the treatment investigated may be challenging as the time since discontinuation increases [13]. Exacerbating this challenge may be the possible receipt of subsequent therapy by patients who have discontinued. Interpreting PRO data and particularly PRO tolerability data when subsequent treatments have commenced may be difficult. Furthermore, patients who may have discontinued trial therapy and identified another trial for their indication may be completing PROs for that trial, which could affect completion rates for post-discontinuation evaluation for the original trial. These complexities should be considered by constituents when planning measurement strategies, and a tailored approach can be used when it is known that therapy will likely have long term, irreversible side effects (such as neuropathy).

This study had several limitations. We used data from solid tumour commercial clinical trials, and thus our findings may not necessarily generalise to trials in other settings or patients in cancer clinical care. Although we excluded patients who left treatment due to death from our analysis, it is possible that patients may have died prior to the follow-up time point and thus the completion rates could be underestimated. The analyses presented in this paper use descriptive statistics, without attempts to mitigate missing data. As such, the analyses in the paper may be subject to bias due to missing data. Lastly, the symptom items selected (lack of energy, pain, fatigue, nausea) were not specified as disease-related or symptomatic side effects. In practice, such classification may be difficult for some such items; pain, for example, may be a disease-related symptom and a symptomatic side effect, depending on the indication.

## Conclusions

In conclusion, we found that PRO completion at discontinuation and follow-up was variable and tended to be suboptimal at follow-up; variable completion was seen for all evaluated PROs and not specific to the GP5. Additionally, a subset of patients had persistent side effect bother at discontinuation and follow-up. Our findings of the association between GP5 and other PROs suggests that GP5 may be a helpful measure in capturing post-treatment tolerability in some cases. Improving PRO completion at discontinuation may enhance the usefulness of post-treatment tolerability data particularly when the long-term side effects of anti-cancer therapy need further evaluation.

## Data Availability

The data used in this research are not able to be shared as they come from commercial cancer trials and were accessed through the data sharing service Vivli through a data use agreement.

## Appendices

See Appendix Tables 8, 9, 10 and 11.

**Table 8:** Additional symptom item within strata of GP5: AXIS trials

		AXIS2				AXIS1			
GP5 group		GP5 0–1	GP5 0–2	GP5 2–4	GP5 3–4	GP5 0–1	GP5 0–2	GP5 2–4	GP5 3–4
EOT	PRO score	*N* = *122*	*N* = *215*	*N* = *160*	*N* = *67*	*N* = *45*	*N* = *72*	*N* = *40*	*N* = *13*
*HI7 Fatigue*	0–1	77 (63.1)	109 (50.7)	48 (30.2)	16 (24.2)	28 (62.2)	34 (47.2)	8 (20.0)	2 (15.4)
EOT Early		*N* = *36*	*N* = *69*	*N* = *69*	*N* = *36*	*N* = *5*	*N* = *9*	*N* = *4*	*N* = *0*
*HI7 Fatigue*	0–1	23 (63.9)	35 (50.7)	25 (36.8)	13 (37.1)	2 (40.0)	3 (33.3)	1 (25.0)	0
FU		*N* = *61*	*N* = *79*	*N* = *32*	*N* = *14*	*N* = *19*	*N* = *27*	*N* = *12*	*N* = *4*
*HI7 Fatigue*	0–1	45 (73.8)	50 (63.3)	8 (25.0)	3 (21.4)	13 (68.4)	15 (55.6)	2 (16.7)	0

**Table 9:** Additional symptom item and bother item within strata of GP5: PALOMA trial

GP5 group		GP5 0–1	GP5 0–2	GP5 2–4	GP5 3–4
EOT	PRO score	*N* = *192*	*N* = *241*	*N* = *81*	*N* = *32*
*GP2 Nausea*	0–1	178 (92.7)	209 (87.1)	52 (65.0)	21 (65.6)
*B5 Hair Loss Bother*	0–1	150 (79.4)	177 (74.7)	45 (57.0)	18 (58.1)
EOT early	PRO score	*N* = *37*	*N* = *48*	*N* = *21*	*N* = *10*
*GP2 Nausea*	0–1	35 (94.6)	41 (85.4)	12 (57.1)	6 (60.0)
*B5 Hair Loss Bother*	0–1	27 (77.1)	35 (76.1)	13 (61.9)	5 (50.0)
FU	PRO score	*N* = *34*	*N* = *39*	*N* = *11*	*N* = *6*
*GP2 Nausea*	0–1	34 (100.0)	37 (94.9)	6 (54.5)	3 (50.0)
*B5 Hair Loss Bother*	0–1	30 (88.2)	34 (87.2)	6 (60.0)	2 (40.0)

**Table 10:** Visit and completion rates: ‘expected’ as denominator

Trial	AXIS2	AXIS1	PALOMA
*Discontinuation visit (Any)*			
visit expected	379	121	340
Visit seen	378 (99.7)	108 (89.3)	321 (94.4)
Has GP5	282 (74.4)	85 (70.3)	273 (80.3)
*Discontinuation visit (Early)*			
Visit expected	155	16	73
Visit Seen	155 (100)	14 (87.5)	71 (97.3)
Has GP5	105 (67.7)	9 (56.3)	58 (79.5)
*Follow-up visit*			
Visit expected	183	45	203
Visit seen	171 (93.4)	40 (88.9)	86 (42.4)
Has GP5	93 (50.8)	31 (68.9)	45 (22.2)

**Table 11:** Completion rates, stratified by discontinuation reason*

	AXIS2		AXIS1		PALOMA	
	Progression	Toxicity	Progression	Toxicity	Progression	Toxicity
*Discontinuation (Any)*	*N* = *324*	*N* = *54*	*N* = *98*	*N* = *10*	*N* = *288*	*N* = *33*
Usual activities	255 (78.7)	33 (61.1)	80 (81.6)	5 (50.0)	259 (89.9)	18 (54.5)
Self-care	255 (78.7)	33 (61.1)	79 (80.6)	5 (50.0)	258 (89.6)	16 (48.5)
Mobility	255 (78.7)	34 (63.0)	79 (80.6)	5 (50.0)	259 (89.9)	16 (48.5)
VAS	252 (77.8)	34 (63.0)	80 (81.6)	5 (50.0)	256 (88.9)	17 (51.5)
GP1 Lack Energy	253 (78.1)	34 (63.0)	80 (81.6)	6 (60.0)	258 (89.6)	17 (51.5)
GP4 Pain	252 (77.8)	34 (63.0)	80 (81.6)	6 (60.0)	255 (88.5)	17 (51.5)
GP5 Side effect bother	248 (76.5)	34 (63.0)	80 (81.6)	5 (50.0)	257 (89.2)	16 (48.5)
HI7 Fatigue	253 (78.1)	33 (61.1)	80 (81.6)	6 (60.0)	NA	NA
GP2 Nausea	NA	NA	NA	NA	256 (88.9)	17 (51.5)
B5 Hair loss bother	NA	NA	NA	NA	253 (87.8)	17 (51.5)
*Discontinuation (early)*	*N* = *123*	*N* = *32*	*N* = *13*	*N* = *1*	*N* = *59*	*N* = *12*
Usual activities	87 (70.7)	21 (65.6)	9 (69.2)	0 (0.0)	51 (86.4)	8 (66.7)
Self-care	87 (70.7)	21 (65.6)	8 (61.5)	0 (0.0)	50 (84.7)	7 (58.3)
Mobility	87 (70.7)	22 (68.8)	8 (61.5)	0 (0.0)	51 (86.4)	7 (58.3)
VAS	87 (70.7)	22 (68.8)	9 (69.2)	0 (0.0)	51 (86.4)	7 (58.3)
GP1 Lack energy	86 (69.9)	22 (68.8)	9 (69.2)	0 (0.0)	51 (86.4)	7 (58.3)
GP4 pain	85 (69.1)	22 (68.8)	9 (69.2)	0 (0.0)	51 (86.4)	7 (58.3)
GP5 Side effect bother	83 (67.5)	22 (68.8)	9 (69.2)	0 (0.0)	51 (86.4)	7 (58.3)
HI7 Fatigue	86 (69.9)	21 (65.6)	9 (69.2)	0 (0.0)	NA	NA
GP2 Nausea	NA	NA	NA	NA	51 (86.4)	7 (58.3)
B5 Hair loss bother	NA	NA	NA	NA	49 (83.1)	7 (58.3)
*Follow-up*	*N* = *146*	*N* = *25*	*N* = *38*	*N* = *2*	*N* = *79*	*N* = *7*
Usual activities	81 (55.5)	13 (52.0)	29 (76.3)	2 (100.0)	NA	NA
Self-care	80 (54.8)	13 (52.0)	29 (76.3)	2 (100.0)	NA	NA
Mobility	81 (55.5)	13 (52.0)	29 (76.3)	2 (100.0)	NA	NA
VAS	79 (54.1)	13 (52.0)	29 (76.3)	2 (100.0)	NA	NA
GP1 Lack energy	84 (57.5)	13 (52.0)	29 (76.3)	2 (100.0)	43 (54.4)	3 (42.9)
GP4 pain	83 (56.8)	13 (52.0)	29 (76.3)	2 (100.0)	43 (54.4)	3 (42.9)
GP5 Side effect bother	82 (56.2)	11 (44.0)	29 (76.3)	2 (100.0)	42 (53.2)	3 (42.9)
HI7 Fatigue	84 (57.5)	13 (52.0)	29 (76.3)	2 (100.0)	NA	NA
GP2 Nausea	NA	NA	NA	NA	43 (54.4)	3 (42.9)
B5 Hair loss bother	NA	NA	NA	NA	42 (53.2)	4 (57.1)

*Number with visit as denominator
